# Speeding up respiratory diagnosis: clinical drivers, barriers, and system readiness for rapid molecular point-of-care testing in Saudi Arabia

**DOI:** 10.3389/fpubh.2026.1871669

**Published:** 2026-08-11

**Authors:** Najim Z. Alshahrani, Mohammed AlQahtani, Amal M. Alshahrani, Abdulrahman Alanazi, Nesrin Alharthy, Norah Albalawi, Tareq Khawaji, Weam Qutub, Mohammed A. Alnamakani, Ezzuddin A. Okmi, Amjad M. Alotaibi, Wesam Bafakih, Mohammed Assiri

**Affiliations:** 1 Department of Family and Community Medicine, Faculty of Medicine, University of Jeddah, Jeddah, Saudi Arabia; 2 Microbiology Section, Pathology Medicine Laboratory, Blood Banks Department, Security Forces Hospital, Riyadh, Saudi Arabia; 3 Public Health Authority, Riyadh, Saudi Arabia; 4 Department of Pathology and Laboratory Medicine, King Abdulaziz Medical City, Ministry of National Guard Health Affairs, Riyadh, Saudi Arabia; 5 Pediatric Emergency Department, King Abdulaziz Medical City, King Saud bin Abdulaziz University for Health Sciences, Riyadh, Saudi Arabia; 6 King Abdullah International Medical Research Center, Ministry of National Guard Health Affairs, Riyadh, Saudi Arabia; 7 Point of Care Section, Laboratory Department, Dr. Sulaiman Al Habib Medical Services Group, Riyadh, Saudi Arabia; 8 Jazan Regional Laboratory, Jazan, Saudi Arabia; 9 Geriatric Center, King Abdulaziz Hospital, Makkah Health Cluster, Makkah, Saudi Arabia; 10 Dr. Sulaiman Al Habib Medical Services Group, Riyadh, Saudi Arabia; 11 Tuberculosis and Respiratory Infection Surveillance and Prevention, Public Health Authority, Riyadh, Saudi Arabia; 12 Point of Care Testing, Department of Pathology and Laboratory Medicine, King Faisal Specialist Hospital & Research Centre, Riyadh, Saudi Arabia; 13 Point of Care Testing Department, Jeddah Second Cluster, Ministry of Health, Jeddah, Saudi Arabia; 14 Medical Laboratory and Blood Bank Department, Maternity and Children Hospital, Ministry of Health, Abha, Saudi Arabia

**Keywords:** antimicrobial stewardship (AMS), COVID-19, diagnostics, influenza, molecular point-of-care testing, POCT (point-of-care testing), rapid molecular diagnostics, respiratory infections

## Abstract

**Background:**

Acute respiratory tract infections place substantial strain on healthcare systems, particularly during seasonal respiratory surges and mass-gathering events. Delayed confirmation of respiratory pathogens contributes to diagnostic uncertainty, unnecessary antimicrobial use, delayed infection prevention decisions, and inefficient patient flow. Rapid molecular point-of-care testing (mPOCT) offers near-PCR-level diagnostic accuracy with turnaround times compatible with real-time clinical decision-making; however, implementation and integration into routine clinical pathways remain variable across healthcare systems.

**Methods:**

We conducted an implementation-focused mixed-methods descriptive study integrating a synthesis of published evidence with structured stakeholder engagement in Saudi Arabia. Primary data were collected through three multidisciplinary scientific meetings and a stakeholder survey (*N* = 35) involving physicians, point-of-care experts, and laboratory professionals from Ministry of Health (MOH), institutional, and private healthcare settings. Quantitative survey data were analyzed descriptively using R version 4.3.3, including exploratory descriptive subgroup summaries by workplace sector and stakeholder role. Qualitative findings from expert discussions were synthesized thematically and integrated with survey findings and published evidence through methodological triangulation.

**Results:**

Physicians represented 48.6% of respondents, with most participants working in MOH facilities (60.0%). Rapid mPOCT was reported to be routinely used during respiratory seasons, with 68.6% indicating daily use. Immediate treatment decision-making (74.3%) and patient management activities, including bed designation (57.1%), were the most frequently reported indications for testing. Nearly half of respondents preferred results within 15 min (45.7%), while most preferred turnaround times of 30 min or less (85.7%). Participants reported generally moderate-to-high implementation of rapid mPOCT (mean implementation score 7.69/10; range 2–10), although variability remained in guideline integration, standard operating procedures, and institutional implementation. Frequently reported implementation barriers included limited education or guidance (31.4%), perceived availability of alternative diagnostic approaches (28.6%), and financial or reimbursement constraints (28.6%).

**Conclusion:**

Saudi healthcare stakeholders perceived rapid mPOCT as a valuable diagnostic tool for respiratory infections, particularly in emergency and acute care settings where timely results may support clinical decision-making. Wider implementation will require standardized diagnostic pathways, workforce education, supportive governance, and sustainable reimbursement strategies to facilitate consistent integration into routine clinical practice.

## 1 Background

Acute respiratory tract infections (RTIs) remain among the leading causes of morbidity and mortality worldwide and place substantial pressure on healthcare systems, particularly during seasonal peaks (1). Respiratory viruses account for a large proportion of microbiologically confirmed RTIs, with influenza viruses, respiratory syncytial virus (RSV), and SARS-CoV-2 representing key pathogens across age groups (2). Despite the viral predominance of many clinical presentations, antibiotic prescribing remains common because of diagnostic uncertainty, contributing to inappropriate antimicrobial use and accelerating antimicrobial resistance (3).

Conventional diagnostic pathways rely on centralized laboratory testing, including polymerase chain reaction (PCR) and culture-based methods (4). Although these approaches provide high analytical performance, they are frequently associated with delays in turnaround time, particularly in high-volume settings or when samples require transport to referral laboratories (5). Such delays can limit timely clinical decision-making, lead to empirical treatment, delay infection control measures, prolong emergency department stays, and result in inefficient bed utilization (5). Consequently, diagnostic strategies that support same-encounter decision-making are increasingly needed to optimize patient management and strengthen diagnostic and antimicrobial stewardship (6).

Rapid molecular point-of-care testing (mPOCT) has emerged as a key innovation, combining near-PCR-level accuracy with results available within minutes (7). These platforms enable immediate treatment decisions, improve patient flow, and enhance infection prevention and control through earlier identification of transmissible pathogens (7). Evidence from diverse clinical settings indicates that rapid molecular testing can improve targeted antiviral prescribing, reduce unnecessary antibiotic exposure, and strengthen respiratory pathogen surveillance (7). However, real-world implementation remains heterogeneous owing to operational, financial, and health system–level factors, including variability in test placement, staff training, governance models, and the availability of standardized clinical algorithms (7).

Saudi Arabia represents a particularly relevant setting for evaluating rapid molecular point-of-care testing (mPOCT) because of its advanced healthcare infrastructure, ongoing investments in diagnostic innovation, and distinctive respiratory epidemiology shaped by seasonal respiratory infections and large-scale mass gatherings, including Hajj and Umrah (8). Despite these advantages, variability remains in the integration of rapid molecular diagnostics into routine clinical workflows, standardized diagnostic pathways, and institutional implementation strategies.

To address these gaps, this implementation-focused mixed-methods study integrates evidence from the published literature with a multidisciplinary Saudi stakeholder survey and qualitative expert discussions to evaluate stakeholder perceptions, implementation readiness, and health system considerations for rapid molecular point-of-care testing for respiratory infections. By triangulating published evidence with real-world implementation perspectives, the study aims to identify context-specific opportunities and barriers that may inform future clinical practice, health system planning, and policy development in Saudi Arabia.

## 2 Methods

### 2.1 Study design

This study employed an implementation-focused mixed-methods descriptive design integrating quantitative and qualitative stakeholder data with a targeted narrative review of the published literature to evaluate the clinical drivers, implementation barriers, and health system readiness for rapid molecular point-of-care testing (mPOCT) for respiratory infections in Saudi Arabia. The study was designed to provide a pragmatic assessment of current implementation practices and inform context-specific recommendations for integrating rapid molecular diagnostics into respiratory care pathways rather than to evaluate the diagnostic accuracy, clinical effectiveness, or cost-effectiveness of specific testing platforms.

Three complementary sources of evidence were incorporated within a convergent mixed-methods framework: (1) a targeted narrative review of published evidence describing the clinical, operational, and health system implications of respiratory mPOCT; (2) a structured stakeholder survey capturing current implementation practices, perceptions, and priorities among healthcare professionals involved in respiratory diagnostics; and (3) qualitative data derived from multidisciplinary scientific meetings exploring implementation experiences, perceived barriers, and future policy directions.

**Figure 1:**
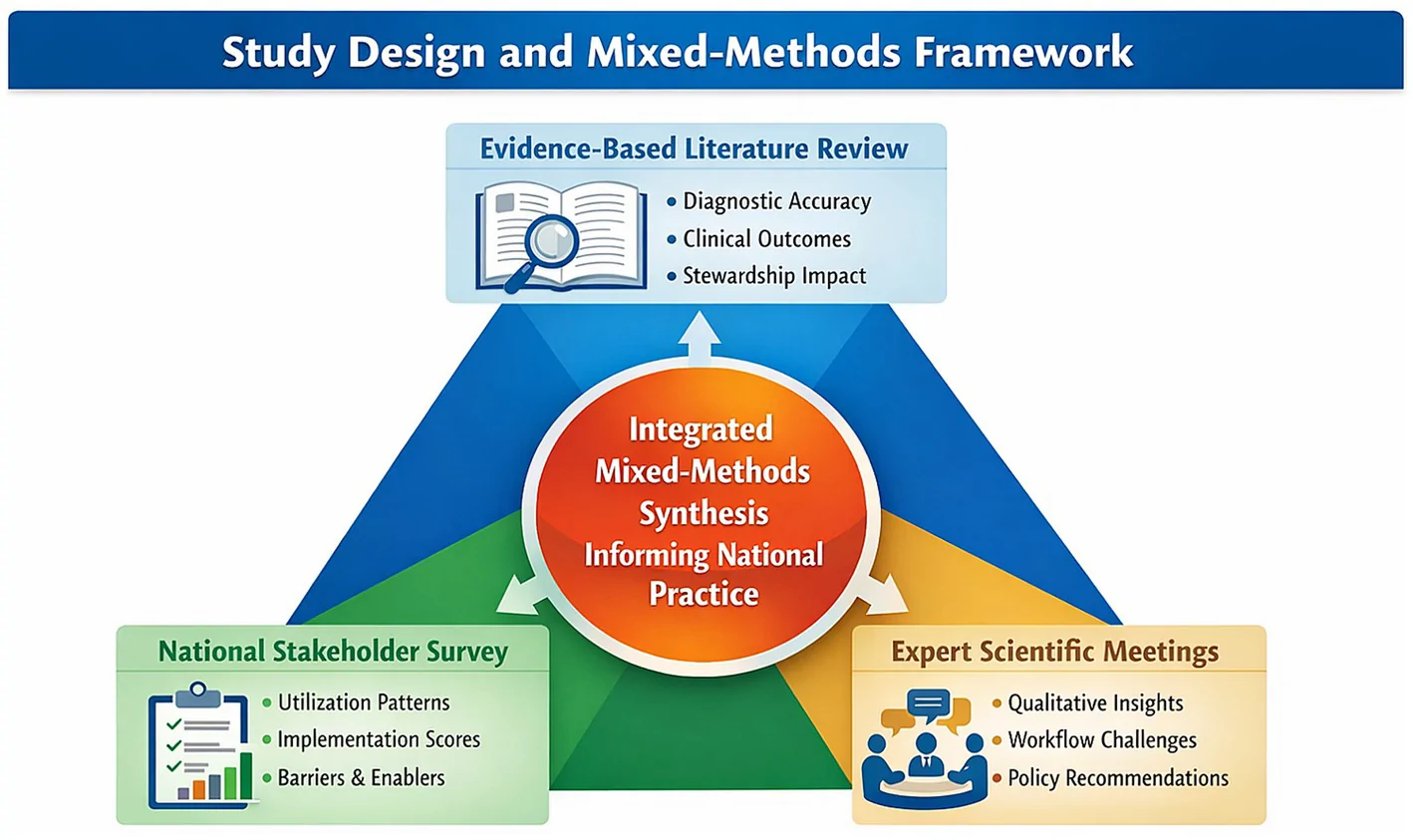
Study design and mixed-methods framework. Schematic overview of the study design integrating an evidence-based narrative review, a national stakeholder survey, and qualitative expert discussions conducted across three scientific meetings to evaluate the clinical value and implementation of rapid molecular point-of-care testing for respiratory infectious diseases in Saudi Arabia.

These three evidence streams were analyzed independently and subsequently integrated through methodological triangulation to identify areas of convergence and divergence between published evidence and real-world stakeholder perspectives. The integrated synthesis informed interpretation of implementation readiness, identification of priority barriers, and development of expert-informed conceptual diagnostic pathways for respiratory mPOCT within Saudi healthcare settings. The overall methodological framework and evidence integration process are illustrated in Figure 1.

### 2.2 Study setting and stakeholder recruitment

Primary data collection was conducted during three multidisciplinary scientific meetings held in Riyadh, Saudi Arabia, in 2025. The meetings were organized to facilitate structured discussion regarding the implementation of rapid molecular point-of-care testing for respiratory pathogens across different healthcare settings within the Kingdom.

The meetings brought together healthcare professionals with expertise or operational responsibility in respiratory diagnostics, point-of-care testing, laboratory medicine, infectious diseases, emergency medicine, family medicine, pediatrics, infection prevention and control, healthcare management, and diagnostic implementation. Participants represented a range of healthcare sectors, including Ministry of Health (MOH) hospitals, governmental and institutional healthcare organizations, and private healthcare providers.

Eligible stakeholders were identified before each meeting based on their professional expertise, leadership roles, or practical experience in respiratory diagnostic services and point-of-care testing implementation. Invitations were distributed before the scientific meetings through the organizing committee. Participation in both the meetings and the accompanying survey was voluntary. No financial incentives were provided to participants for survey completion or meeting participation.

Although participants represented multiple healthcare sectors and professional disciplines, recruitment was centered around scientific meetings conducted in Riyadh. Accordingly, this study is presented as a multidisciplinary Saudi stakeholder assessment rather than a nationally representative survey of all healthcare professionals.

### 2.3 Participant selection and sampling strategy

A purposive expert sampling strategy was used to recruit participants capable of providing informed perspectives on the implementation of respiratory molecular point-of-care testing within Saudi healthcare settings. The sampling approach was intentionally designed to capture a broad range of implementation experiences rather than to estimate population-level parameters.

Eligible participants included physicians involved in respiratory patient management, laboratory specialists, molecular microbiologists, point-of-care testing leaders, infection prevention specialists, healthcare administrators, and professionals responsible for diagnostic service planning or implementation. Participants were selected based on their recognized professional expertise, institutional responsibilities, or direct involvement in respiratory diagnostic pathways.

Healthcare professionals attending the scientific meetings were invited to complete a structured stakeholder questionnaire. Participation was entirely voluntary, anonymous, and independent of meeting participation. Completion of the questionnaire implied informed consent for the anonymous use of aggregated responses for research purposes.

A total of 35 stakeholders completed the questionnaire and constituted the final analytical sample. Because recruitment occurred through invitation to multidisciplinary scientific meetings rather than systematic sampling from a predefined sampling frame, a formal response rate could not be calculated. Consequently, findings should be interpreted as reflecting expert stakeholder perspectives rather than nationally representative estimates of clinical practice across Saudi Arabia.

### 2.4 Scientific meetings

Three structured scientific meetings were conducted using a standardized discussion agenda developed before study initiation to ensure consistency across all sessions. The meetings focused on the current use of rapid molecular POCT, implementation experiences, operational challenges, diagnostic stewardship, workforce preparedness, clinical workflow integration, reimbursement considerations, and future priorities for respiratory diagnostic services.

Each meeting was facilitated by experienced moderators using semi-structured discussion prompts to encourage open multidisciplinary discussion while maintaining consistency in the topics explored. Discussions emphasized practical implementation experiences rather than evaluation of individual commercial diagnostic platforms.

Key discussion points and consensus statements were documented contemporaneously by designated rapporteurs using structured meeting notes. Following completion of each meeting, the notes were reviewed by members of the study team to ensure completeness and consistency before qualitative analysis. These documented discussions formed the qualitative dataset used for thematic synthesis.

The scientific meetings also provided an opportunity to review preliminary survey findings, discuss areas of agreement and disagreement, and identify implementation priorities relevant to Saudi healthcare practice. However, quantitative survey responses and qualitative discussion data were analyzed separately before integration during the triangulation stage.

### 2.5 Survey development

A structured stakeholder questionnaire was specifically developed for this implementation study following review of the published literature on respiratory molecular point-of-care testing, diagnostic stewardship, implementation science, and healthcare workflow optimization. Questionnaire development was additionally informed by the predefined objectives of the scientific meetings and expert input from clinicians, laboratory specialists, and point-of-care implementation leaders participating in study planning.

The questionnaire comprised predominantly closed-ended questions designed to characterize current implementation practices and stakeholder perceptions rather than to evaluate clinical outcomes. Survey domains included participant demographics and professional role; healthcare sector; previous experience with rapid molecular POCT; testing workflows and clinical settings; availability of respiratory diagnostic algorithms and standard operating procedures; perceived implementation maturity; clinical indications for testing; preferred respiratory pathogens for molecular testing; desired turnaround time; implementation barriers; perceived clinical value; and perceptions regarding operational and economic utility.

Questions involving implementation barriers, preferred pathogens, and perceived clinical value permitted multiple responses where appropriate. Overall implementation readiness was assessed using a self-reported numerical rating scale ranging from 0 (no implementation) to 10 (fully implemented). The complete questionnaire is provided as Supplementary File S1.

The survey was administered immediately following the scientific meetings using a standardized format. Responses were anonymized before analysis, and no personally identifiable information was collected. Data were reviewed for completeness prior to consolidation into a single analytical dataset.

### 2.6 Narrative literature review

A targeted narrative review was undertaken to provide contemporary scientific context for interpreting stakeholder perspectives and to support evidence triangulation within the mixed-methods framework. The review was intended to summarize current knowledge regarding the implementation and clinical application of rapid molecular point-of-care testing rather than to conduct a systematic evidence synthesis or meta-analysis.

Structured literature searches were performed using PubMed and Scopus, supplemented by manual screening of reference lists from relevant systematic reviews, international guidance documents, and key implementation reports. The review primarily considered publications published during the preceding 10–15 years, although landmark studies and foundational guidance documents were also included where appropriate.

Evidence was prioritized according to its relevance to implementation of respiratory mPOCT and included systematic reviews, randomized controlled trials, observational studies, implementation evaluations, health systems research, and international recommendations. Particular emphasis was placed on studies addressing diagnostic stewardship, antimicrobial stewardship, emergency department workflow, infection prevention and control, operational implementation, healthcare resource utilization, and health economic considerations for influenza A/B, respiratory syncytial virus (RSV), SARS-CoV-2, and Group A Streptococcus.

**Figure 2:**
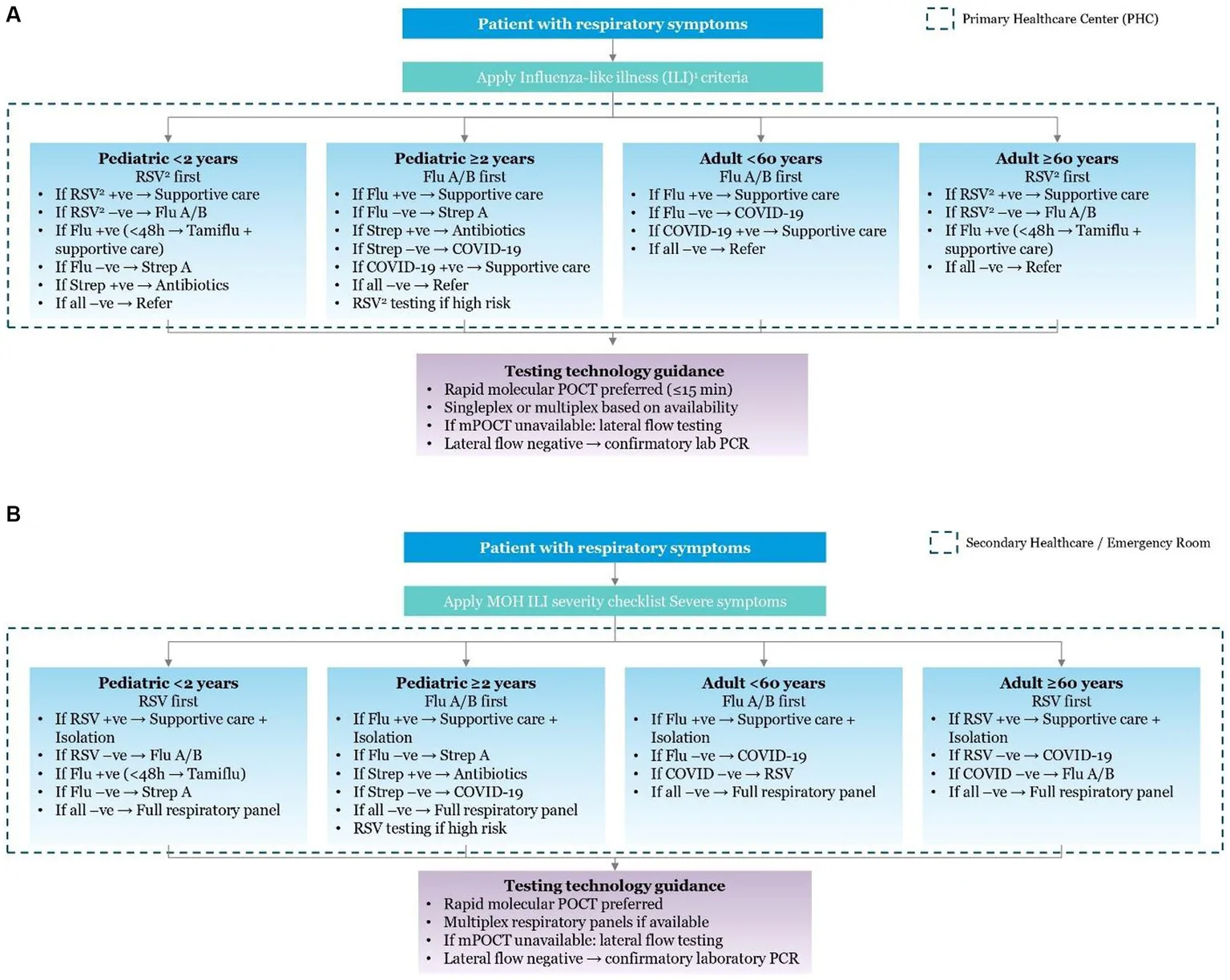
Clinical and operational algorithm for rapid molecular point-of-care testing (mPOCT) in acute respiratory presentations. **(A)** Diagnostic pathway for patients with mild to moderate respiratory symptoms managed in primary healthcare settings. This diagnostic algorithm was developed based on currently available local respiratory sentinel surveillance data from Saudi Arabia. Diagnostic testing and treatment decisions for respiratory infections should be guided by local epidemiology, circulating respiratory pathogens, seasonality, and antimicrobial resistance patterns, and should be adapted as surveillance data and national guidance evolve. The upper age limit for pediatric populations may vary according to national regulations. While the World Health Organization (WHO) defines pediatric populations as individuals up to 18 years of age, local policies and clinical practice may apply different age thresholds. ^1^Influenza-like illness (ILI) is defined according to the World Health Organization (WHO) case definition as an acute respiratory infection with measured fever ≥38 °C and cough, with symptom onset within the previous 10 days. ^2^Respiratory syncytial virus (RSV) high-risk populations for severe disease requiring hospitalization include infants under 6 months of age, premature infants, children with chronic lung or congenital heart disease, older adults (≥60–65 years), and immunocompromised individuals. **(B)** Diagnostic pathway for patients with severe respiratory symptoms managed in secondary and emergency care settings. Rapid molecular POCT is the preferred first-line modality where available; lateral flow testing may be used as an alternative with confirmatory laboratory testing for negative results. This diagnostic algorithm was developed based on currently available local respiratory sentinel surveillance data from Saudi Arabia. Diagnostic testing and treatment decisions for respiratory infections should be guided by local epidemiology, circulating respiratory pathogens, seasonality, and antimicrobial resistance patterns, and should be adapted as surveillance data and national guidance evolve. The upper age limit for pediatric populations may vary according to national regulations. While the World Health Organization (WHO) defines pediatric populations as individuals up to 18 years of age, local policies and clinical practice may apply different age thresholds. ^1^Severe acute respiratory infection (SARI) is defined according to the World Health Organization (WHO) case definition as an acute respiratory infection with a history of fever or measured fever ≥38 °C and cough, with symptom onset within the previous 10 days, requiring hospitalization. ^2^Respiratory syncytial virus (RSV) high-risk populations for severe disease requiring hospitalization include infants under 6 months of age, premature infants, children with chronic lung or congenital heart disease, older adults (≥60–65 years), and immunocompromised individuals.

The literature review served exclusively as contextual evidence for interpretation of stakeholder findings and was not used to generate pooled estimates of diagnostic performance, clinical effectiveness, antimicrobial impact, or economic benefit. Instead, published evidence was subsequently integrated with quantitative survey findings and qualitative stakeholder insights through methodological triangulation to identify consistent implementation themes and inform development of the proposed expert-informed respiratory diagnostic pathways presented in Figure 2. These diagnostic pathways represent conceptual implementation frameworks derived from the combined evidence sources and expert consensus and have not been prospectively validated or formally evaluated for clinical effectiveness.

### 2.7 Quantitative analysis

Survey data were analyzed using R statistical software (version 4.3.3; R Foundation for Statistical Computing, Vienna, Austria) using tidyverse-based analytical workflows. Given the implementation-focused and exploratory nature of the study, all quantitative analyses were prespecified as descriptive. Categorical variables were summarized as frequencies and percentages. For survey items permitting multiple responses, each response option was treated independently and summarized as the number and proportion of participants selecting that option. Continuous and ordinal variables, including the self-reported implementation readiness score (0–10 scale), were summarized using the mean, standard deviation (SD), median, interquartile range (IQR), minimum, and maximum values, as appropriate.

Exploratory descriptive subgroup summaries were generated according to workplace sector (Ministry of Health versus non-Ministry of Health), stakeholder category (clinical versus non-clinical), and previous experience with rapid molecular POCT. These subgroup summaries were intended solely to describe variation in stakeholder responses and implementation patterns across participant groups. No formal hypothesis testing, statistical significance testing, or multivariable modeling was planned because of the modest sample size and the purposive sampling strategy. Accordingly, subgroup findings should be interpreted as descriptive patterns that may generate hypotheses for future studies rather than as evidence of statistically significant differences between groups. Missing responses were reported where applicable, and percentages were calculated using the number of participants responding to each survey item as the denominator.

### 2.8 Qualitative thematic analysis

Qualitative data were derived from the multidisciplinary scientific meetings conducted as part of stakeholder engagement. Discussions were documented using structured meeting notes prepared during each session by designated rapporteurs following a predefined discussion agenda. The qualitative component was intended to capture implementation experiences, operational challenges, and expert perspectives regarding the integration of rapid molecular POCT into routine respiratory care rather than to explore individual lived experiences.

Qualitative data were analyzed using an inductive thematic content analysis approach. Following completion of the meetings, documented discussion notes from all three sessions were reviewed repeatedly by members of the study team to achieve familiarity with the data. Recurring concepts, implementation challenges, recommendations, and areas of consensus were identified through iterative coding and subsequently grouped into broader thematic domains.

Themes were refined through discussion among the investigators until consensus was achieved regarding their interpretation and organization. The final thematic framework focused on three overarching implementation domains: (1) perceived clinical value of rapid molecular POCT, including timely clinical decision-making, patient management, and diagnostic stewardship; (2) operational implementation challenges, including workflow integration, workforce training, test placement, and resource constraints; and (3) health system priorities, including governance, standardized clinical pathways, reimbursement considerations, and long-term sustainability.

To enhance credibility, qualitative findings were interpreted alongside survey results and published evidence rather than in isolation. Representative statements illustrating major themes are presented in the qualitative summary table (Table 1). The qualitative synthesis was intended to identify common implementation priorities and contextualize quantitative findings rather than quantify stakeholder agreement or generate formal qualitative theory. The scientific meetings were conducted to facilitate multidisciplinary discussion rather than formal qualitative interviews. Consequently, the qualitative findings should be interpreted as expert implementation perspectives generated within a structured scientific meeting environment.

**Table 1:** Qualitative thematic framework derived from multidisciplinary stakeholder discussions.

Major theme	Subtheme	Supporting evidence from stakeholder discussions	Representative stakeholder statement*
Clinical value of rapid molecular POCT	Timely clinical decision-making	Participants consistently emphasized that rapid molecular POCT provides the greatest value when results are available during the same clinical encounter, allowing immediate treatment and disposition decisions.	*“Rapid results are clinically valuable only if they are available early enough to influence management decisions during the patient’s visit.”*
	Patient management and triage	Rapid molecular testing was viewed as supporting patient placement, admission decisions, isolation, and appropriate bed designation, particularly during respiratory seasons.	*“Early identification of respiratory pathogens improves patient flow and facilitates appropriate placement and isolation.”*
	Diagnostic stewardship	Participants considered rapid molecular POCT an important component of evidence-based diagnostic pathways that reduces diagnostic uncertainty when integrated into clinical algorithms.	*“Testing should be performed when it will change management rather than as a routine investigation.”*
Operational implementation challenges	Workflow integration	Successful implementation was considered dependent on integrating testing into existing emergency department and outpatient workflows rather than simply introducing new diagnostic devices.	*“Implementation requires redesigning clinical workflows, not simply purchasing equipment.”*
	Workforce education and competency	Participants identified insufficient education regarding test indications, interpretation, and clinical application as a major implementation barrier.	*“Healthcare professionals need guidance on when to test, how to interpret results, and how findings should influence clinical decisions.”*
	Test placement	Stakeholders emphasized that placing instruments in true point-of-care settings maximizes clinical benefit compared with centralized laboratory placement.	*“The greatest value is achieved when testing occurs where clinical decisions are made.”*
	Resource and reimbursement constraints	Financial limitations, reimbursement uncertainty, and infrastructure requirements were commonly discussed as barriers to wider implementation.	*“Sustainable implementation requires adequate funding and institutional support.”*
Health system priorities	Standardized clinical pathways	Participants strongly supported development of national respiratory testing algorithms to improve consistency and reduce variation across institutions.	*“National algorithms would help standardize testing and reduce unnecessary practice variation.”*
	Governance and quality assurance	Stakeholders highlighted the importance of clear governance structures, SOPs, quality assurance, and laboratory oversight for decentralized molecular testing.	*“Implementation should be supported by clear governance, quality assurance, and standardized operating procedures.”*
	Long-term sustainability	Participants emphasized that sustainable implementation requires alignment with national policy, reimbursement mechanisms, antimicrobial stewardship, and digital health infrastructure.	*“Long-term success depends on integrating rapid molecular POCT into broader healthcare system priorities rather than implementing it as a standalone technology.”*

POCT, point-of-care testing; SOP, standard operating procedure. *Representative stakeholder statements are illustrative paraphrased summaries synthesized from multidisciplinary scientific meeting discussions. They are presented to reflect the predominant views expressed across participants and are not verbatim quotations from individual stakeholders.

### 2.9 Evidence integration and algorithm development

Integration of quantitative, qualitative, and literature-derived evidence was undertaken using a convergent triangulation approach. Each evidence source was initially analyzed independently before being compared to identify areas of agreement, complementarity, and divergence regarding the implementation of rapid molecular POCT within Saudi healthcare settings.

The narrative review provided the external scientific evidence describing current knowledge regarding diagnostic stewardship, clinical applications, implementation strategies, and health system considerations. The stakeholder survey characterized current implementation practices, perceived barriers, and operational priorities within Saudi healthcare institutions. Qualitative thematic analysis provided contextual understanding of implementation challenges, workflow considerations, and policy recommendations emerging from multidisciplinary expert discussions.

Evidence from these three complementary sources was subsequently synthesized through investigator consensus to generate an integrated interpretation of implementation readiness and identify practical recommendations for strengthening respiratory diagnostic pathways. Particular emphasis was placed on areas consistently identified across all evidence streams, including the importance of timely testing, standardized clinical algorithms, workforce education, diagnostic stewardship, workflow integration, and supportive governance structures.

Based on this integrated synthesis, conceptual respiratory diagnostic pathways for primary healthcare and secondary/emergency care settings were developed (Figures 2A,B). The proposed pathways were designed to illustrate how rapid molecular POCT may be pragmatically incorporated into existing clinical workflows under Saudi healthcare conditions while reflecting current evidence, stakeholder priorities, and antimicrobial stewardship principles.

The proposed algorithms are expert-informed conceptual implementation frameworks and should not be interpreted as validated clinical guidelines, Ministry of Health recommendations, or evidence-based diagnostic protocols. They have not been prospectively evaluated for diagnostic performance, clinical effectiveness, operational impact, or cost-effectiveness and are intended primarily to support future implementation research, clinical pathway development, and policy discussion.

### 2.10 Ethical considerations

The study was conducted in accordance with the ethical principles of the Declaration of Helsinki and the applicable ethical framework governing research in the Kingdom of Saudi Arabia. Ethical oversight of biomedical research in Saudi Arabia is governed by the National Committee of Bioethics (NCBE), established under King Abdulaziz City for Science and Technology (KACST), which develops national standards and regulations for research involving living subjects and oversees their implementation through registered local ethics committees. Participation in both the scientific meetings and the stakeholder survey was entirely voluntary. Before survey administration, participants were informed of the study objectives, the anonymous nature of data collection, the intended use of aggregated findings for research and publication purposes, and their right to decline participation without consequence. Completion of the anonymous questionnaire was considered to constitute informed consent for participation.

The study did not involve patients, biological specimens, clinical interventions, or identifiable personal information. Data collection was limited to anonymous professional opinions and implementation experiences provided by healthcare professionals. Because the project constituted an implementation-focused stakeholder engagement and health service evaluation activity involving anonymous professional participants and did not involve biomedical research on human participants as defined under the applicable Saudi regulatory framework, formal Institutional Review Board (IRB) approval was not required. All study data were analyzed and reported in aggregate to preserve participant confidentiality.

## 3 Results

### 3.1 Participant characteristics

A total of 35 multidisciplinary stakeholders completed the structured questionnaire and constituted the analytical sample. Physicians represented nearly half of all respondents (17/35, 48.6%), followed by point-of-care testing (POCT) experts (10/35, 28.6%) and laboratory professionals (8/35, 22.9%). Most participants were employed within Ministry of Health (MOH) institutions (21/35, 60.0%), while the remainder represented governmental or institutional hospitals (9/35, 25.7%) and private healthcare organizations (5/35, 14.3%).

Participants reported varying levels of previous experience with rapid molecular POCT, ranging from no previous use to more than five years of experience. Current testing pathways also varied across institutions. Sixteen participants (45.7%) reported that testing was routinely performed in true point-of-care settings, including emergency departments, triage areas, or inpatient wards, whereas others reported laboratory-based POCT (9/35, 25.7%), centralized laboratory testing (6/35, 17.1%), or referral laboratory testing (4/35, 11.4%) (Table 2).

**Table 2:** Participant characteristics and rapid molecular POCT context (*N* = 35).

Characteristic	Category	*n*	%
Professional role	Physician	17	48.6
	POCT expert	10	28.6
	Laboratory expert	8	22.9
Workplace sector	MOH	21	60.0
	Public/Institutional hospital	9	25.7
	Private sector	5	14.3
Experience with rapid molecular POCT	>5 years	11	31.4
	2–5 years	9	25.7
	<2 years	8	22.9
	No prior use	7	20.0
Where testing is performed in practice	ER/triage/wards (true POC setting)	16	45.7
	POC platform located in laboratory	9	25.7
	Central laboratory	6	17.1
	External/referral laboratory	4	11.4

ER, emergency room; MOH, Ministry of Health; POCT, point-of-care testing.

Most respondents (26/35, 74.3%) indicated that a respiratory testing algorithm for influenza, respiratory syncytial virus (RSV), SARS-CoV-2, and Group A Streptococcus was available within their institution (Supplementary Table S1). Nevertheless, the reported location of testing remained heterogeneous across participating institutions (Supplementary Table S2).

### 3.2 Utilization patterns and perceived clinical value of rapid molecular POCT

Rapid molecular POCT was reported to be routinely used during respiratory seasons, with 24 of 35 participants (68.6%) indicating daily use. Weekly use was reported by 17.1% of respondents, whereas monthly or no routine use was less frequently reported (Table 3). Immediate treatment decision-making was the most frequently reported indication for rapid molecular POCT (26/35, 74.3%), followed by patient management activities, including bed designation and allocation (20/35, 57.1%). Other commonly reported indications included emergency department workflow optimization (37.1%), management of diagnostically uncertain patients (37.1%), and the need for reliable molecular confirmation of respiratory pathogens (37.1%).

**Table 3:** Utilization patterns and clinical value of rapid molecular POCT during respiratory season (*N* = 35).

Domain	Item	*n*	%
Frequency of rapid molecular POCT use	Daily	24	68.6
	Weekly	6	17.1
	Monthly	1	2.9
	Never	4	11.4
Primary clinical reasons for using rapid molecular POCT (multiple responses allowed)	Immediate treatment decision-making	26	74.3
	Patient management (e.g., bed designation and allocation)	20	57.1
	Emergency department workflow optimization	13	37.1
	Reliable testing as a key priority	13	37.1
	Unclear clinical diagnosis	13	37.1
	Logistical reasons (e.g., laboratory capacity constraints)	3	8.6
Preferred conditions for rapid molecular POCT (multiple responses allowed)	All respiratory conditions are relevant	18	51.4
	Respiratory syncytial virus (RSV)	13	37.1
	Influenza A and B	12	34.3
	SARS-CoV-2 (COVID-19)	11	31.4
	Group A Streptococcus (Strep A)	7	20.0
Preferred turnaround time (swab to result)	<15 min	16	45.7
	<30 min	14	40.0
	<60 min	4	11.4
	Time is not a major factor	1	2.9

Percentages are calculated using total respondents (*N* = 35). Primary clinical reasons and preferred conditions were multi-response items; therefore, totals may exceed 100%. POCT, point-of-care testing; RSV, respiratory syncytial virus.

Participants expressed a clear preference for short turnaround times. Nearly half of respondents (45.7%) considered a turnaround time of 15 min optimal, while most respondents (85.7%) preferred results to be available within 30 min. Regarding preferred diagnostic targets, more than half of participants (18/35, 51.4%) considered rapid molecular POCT applicable across all respiratory conditions. Among individual pathogens, RSV (37.1%), influenza A/B (34.3%), and SARS-CoV-2 (31.4%) were most frequently identified as priorities for rapid molecular testing (Supplementary Table S3).

Stakeholders also identified several perceived factors contributing to the clinical value of rapid molecular POCT. Speed of testing was the most frequently selected attribute (30/35, 85.7%), followed by patient management and workflow efficiency (22/35, 62.9%), reliable diagnostic performance (19/35, 54.3%), and perceived support for antimicrobial stewardship (16/35, 45.7%) (Supplementary Table S4).

**Figure 3:**
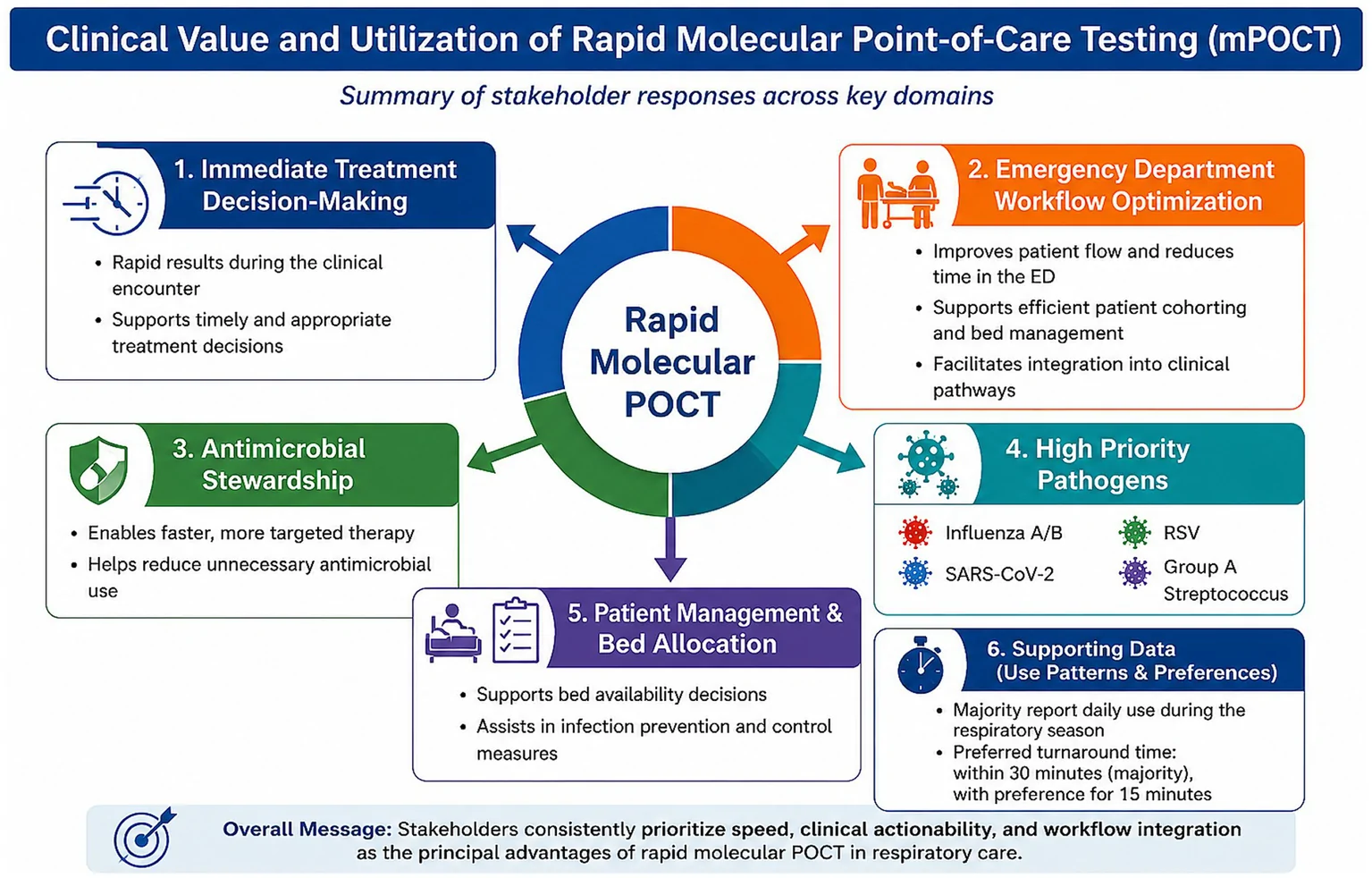
Stakeholder-perceived clinical value and utilization of rapid molecular Point-of-Care Testing (mPOCT) for respiratory infections. This figure summarizes the principal themes identified from the stakeholder survey regarding the perceived clinical value and utilization of rapid molecular point-of-care testing (mPOCT) for respiratory infections. The figure is a conceptual synthesis of stakeholder responses and does not display quantitative survey percentages.

The principal utilization patterns and perceived value domains identified by stakeholders are summarized in Figure 3.

### 3.3 Implementation readiness and health system support

Participants reported generally moderate-to-high implementation of rapid molecular POCT within their institutions, with a mean self-reported implementation score of 7.69 (SD 2.0), a median score of 8, and an observed range from 2 to 10.

Reported implementation barriers varied across respondents. The most frequently reported barrier was limited education or absence of clinical guidance (31.4%), followed by perceived availability of alternative diagnostic approaches (28.6%) and financial or reimbursement constraints (28.6%). Additional barriers included limited test menus and concerns regarding perceived test reliability.

Institutional support for implementation also varied. Many participants reported the availability of standard operating procedures (SOPs), either as general institutional SOPs or platform-specific guidance. However, uncertainty regarding SOP availability remained common. Similarly, respondents reported variability in the incorporation of rapid molecular POCT into institutional or national clinical guidance, with fewer than half indicating formal guideline inclusion and a substantial proportion reporting uncertainty regarding guideline availability (Table 4).

**Table 4:** Implementation status, barriers, and system support for rapid molecular POCT (*N* = 35).

Domain	Item	Value/*n*	%
Implementation level	Mean implementation score (0–10)	7.69	—
	Median implementation score	8	—
	Range	2–10	—
Barriers to full implementation (multiple responses allowed)	Lack of education or guidelines	11	31.4
	Better alternatives perceived/available	10	28.6
	Financial or reimbursement issues	10	28.6
	Limited test parameters/menu	6	17.1
	Concerns about unreliable results	4	11.4
	Not included in guidelines	3	8.6
SOP availability for rapid molecular POCT	Yes, general SOP approach	13	37.1
	Yes, specific technology SOP	12	34.3
	I do not know	7	20.0
	Missing	2	5.7
	No SOPs	1	2.9
Guidelines supporting POCT use	I do not know	10	28.6
	No national guidelines	9	25.7
	Yes, included in hospital protocols	8	22.9
	Yes, included in national guidelines	8	22.9

Implementation score reflects self-rated implementation of rapid molecular POCT (0 = not implemented, 10 = fully implemented). Percentages are calculated using total respondents (*N* = 35). Barriers are multi-response items, so totals may exceed 100%. POCT, point-of-care testing; SOP, standard operating procedure.

When asked to compare testing approaches, respondents most frequently perceived rapid molecular POCT as offering greater clinical value than conventional laboratory PCR (42.9%) or lateral flow testing (34.3%), whereas 20.0% perceived no meaningful difference (Supplementary Table S5).

### 3.4 Qualitative findings from multidisciplinary stakeholder discussions

Qualitative analysis identified three overarching implementation domains: perceived clinical value, operational implementation challenges, and health system priorities. Stakeholders consistently described rapid molecular POCT as most valuable when results were available during the same clinical encounter, particularly in emergency departments, triage areas, and other acute care settings where diagnostic information could directly inform treatment, patient disposition, and infection prevention decisions. The themes identified through thematic analysis were highly consistent across the three scientific meetings, with no major conflicting viewpoints identified among stakeholder groups. Participants also emphasized that successful implementation depends not only on the availability of testing platforms but also on their effective integration into existing clinical workflows.

Workforce education and competency development emerged as recurring implementation priorities. Participants highlighted the need for standardized education addressing appropriate patient selection, interpretation of molecular test results, and incorporation of testing into routine clinical decision-making. Discussions also emphasized the importance of standardized respiratory diagnostic algorithms and clearly defined governance structures to reduce variability in practice across healthcare institutions.

Operational and policy-level challenges, including reimbursement limitations, inconsistent availability of standard operating procedures, variable institutional support, and the absence of harmonized implementation pathways, were repeatedly identified as barriers to wider adoption of rapid molecular POCT. Overall, the qualitative findings complemented the quantitative survey results by providing contextual explanations for the implementation patterns and perceived barriers reported by participants.

### 3.5 Exploratory subgroup patterns

Exploratory descriptive summaries demonstrated variation in stakeholder responses across workplace sectors and professional groups (Table 5). Higher self-reported implementation scores were observed among respondents from non-Ministry of Health institutions than among those working within Ministry of Health facilities. Conversely, respondents from MOH institutions more frequently reported that testing was performed within true point-of-care environments, whereas laboratory-based or centralized workflows were reported more commonly in non-MOH settings.

**Table 5:** Key comparative findings by workplace sector and stakeholder role (*N* = 35).

Comparison domain	Group	*n*	Summary measure	Result
Implementation score (0–10)	MOH	21	Mean (median, range)	7.10 (8, 2–10)
	Non-MOH (Institution + private)	14	Mean (median, range)	8.57 (9, 5–10)
Clear respiratory testing algorithm availability	MOH	21	Yes, *n* (%)	16 (76.2)
			No/Unknown, *n* (%)	5 (23.8)
	Non-MOH (Institution + private)	14	Yes, *n* (%)	10 (71.4)
			No/Unknown, *n* (%)	4 (28.6)
Testing location workflow	MOH	21	True POC setting, *n* (%)	11 (52.4)
			Centralized/other, *n* (%)	10 (47.6)
	Non-MOH (Institution + private)	14	True POC setting, *n* (%)	5 (35.7)
			Centralized/other, *n* (%)	9 (64.3)
Preferred turnaround time	Clinical stakeholders (physicians)	17	<15 min, *n* (%)	6 (35.3)
			≥15 min, *n* (%)	11 (64.7)
	Non-clinical stakeholders (POC + lab experts)	18	<15 min, *n* (%)	10 (55.6)
			≥15 min, *n* (%)	8 (44.4)

“True POC setting” refers to testing performed in emergency departments, triage areas, or clinical wards. “Centralized/other” includes testing performed in laboratories, central laboratories, or external/referral laboratories. ER, emergency room; MOH, Ministry of Health; POC, point-of-care; POCT, point-of-care testing.

Availability of respiratory testing algorithms appeared broadly comparable across workplace sectors. Differences in preferred turnaround time were also observed across professional groups, with non-clinical stakeholders more frequently preferring a turnaround time of 15 min than clinical stakeholders. Because of the exploratory design, purposive sampling strategy, and modest sample size, these subgroup summaries are presented as descriptive patterns only and should not be interpreted as evidence of statistically significant differences between stakeholder groups.

### 3.6 Integrated synthesis

Triangulation of the quantitative survey, qualitative stakeholder discussions, and the narrative literature review identified several implementation themes that were consistently represented across all three evidence sources. These included the importance of rapid turnaround time, integration of molecular POCT into time-sensitive clinical workflows, standardized diagnostic algorithms, workforce education, and supportive governance structures. Educational needs, workflow variability, reimbursement considerations, and inconsistent institutional guidance were identified both in the stakeholder survey and during expert discussions and were similarly reflected within the published implementation literature. Collectively, these convergent findings informed development of the expert-informed conceptual respiratory diagnostic pathways presented in Figure 2.

## 4 Evidence synthesis from published literature

### 4.1 Clinical rationale

Acute respiratory tract infections (ARTIs) remain among the leading causes of outpatient visits, emergency department presentations, hospitalization, and healthcare utilization worldwide (1). Although many respiratory infections are self-limiting, a substantial proportion progress to severe lower respiratory tract disease, particularly among young children, older adults, pregnant women, immunocompromised individuals, and patients with chronic medical conditions (2). Seasonal epidemics of influenza, respiratory syncytial virus (RSV), SARS-CoV-2, and other respiratory pathogens continue to place considerable pressure on healthcare systems through increased demand for diagnostic testing, isolation facilities, inpatient beds, and critical care resources (3). Within Saudi Arabia, the burden of respiratory infections is influenced by unique demographic, environmental, and healthcare system characteristics. The Kingdom hosts a large expatriate workforce originating from numerous countries and experiences substantial international travel throughout the year, facilitating continuous introduction of respiratory pathogens and seasonal viral variants (4).

In addition, respiratory disease transmission is shaped by climatic seasonality and periodic surges in healthcare utilization during winter months, when viral respiratory infections account for a substantial proportion of emergency department visits and hospital admissions (5, 6). These epidemiological characteristics create a clinical environment in which timely differentiation between viral and bacterial respiratory infections is essential for optimizing patient management and healthcare resource utilization.

Despite advances in molecular diagnostics, conventional laboratory-based testing frequently fails to support real-time clinical decision-making. Although laboratory polymerase chain reaction (PCR) assays provide excellent analytical sensitivity and specificity, centralized laboratory workflows often require specimen transport, laboratory processing, quality verification, and electronic reporting before results become available (7–9). Consequently, diagnostic confirmation may occur several hours after the initial clinical encounter, limiting opportunities to influence immediate therapeutic decisions, patient disposition, infection prevention measures, or antimicrobial prescribing. During periods of increased respiratory activity, these delays may contribute to prolonged emergency department stays, inefficient patient cohorting, delayed isolation, and empirical antibiotic prescribing in the absence of microbiological confirmation (10–12).

Rapid molecular point-of-care testing (mPOCT) has emerged as an important strategy to bridge the gap between diagnostic accuracy and clinical timeliness. Unlike conventional laboratory testing, mPOCT platforms employ nucleic acid amplification technologies capable of providing highly sensitive pathogen detection near the point of care within approximately 15–30 min (13). The principal advantage of these technologies lies not solely in their analytical performance but in their ability to deliver actionable diagnostic information during the same clinical encounter. This enables clinicians to incorporate microbiological evidence into decisions regarding antiviral therapy, antimicrobial prescribing, patient isolation, hospital admission, and discharge planning while these decisions remain clinically relevant (14).

Evidence from multiple healthcare settings suggests that the clinical value of rapid molecular diagnostics depends not only on test accuracy but also on integration within existing clinical workflows. Studies have demonstrated that diagnostic interventions are most effective when testing occurs in settings where immediate management decisions are made, including emergency departments, urgent care clinics, acute medical units, and pediatric assessment areas (15, 16). Conversely, when rapid molecular platforms are incorporated into conventional laboratory workflows or operated using batch-processing approaches, much of their potential clinical advantage may be diminished despite excellent diagnostic performance (17).

Rapid molecular testing also supports broader health system objectives beyond individual patient diagnosis. Early identification of respiratory pathogens may facilitate timely implementation of infection prevention and control measures, appropriate patient cohorting, optimization of bed allocation, and more efficient utilization of healthcare resources during seasonal respiratory surges (18–20).

Furthermore, confirmation of viral etiologies may improve diagnostic confidence, thereby supporting more judicious antimicrobial prescribing when combined with appropriate clinical assessment and stewardship interventions (21, 22). Nevertheless, published evidence consistently indicates that diagnostic testing alone is insufficient to improve patient outcomes unless accompanied by standardized clinical pathways, clinician education, and institutional governance that translate test results into appropriate management decisions (23).

Collectively, the published literature supports rapid molecular POCT as a diagnostic strategy with the potential to enhance clinical decision-making and operational efficiency when implemented within integrated care pathways. Rather than functioning solely as a laboratory technology, mPOCT should be considered a health system intervention whose effectiveness depends on timely deployment, multidisciplinary collaboration, and incorporation into evidence-based diagnostic and antimicrobial stewardship frameworks. These considerations provide the clinical rationale for evaluating stakeholder perspectives on implementation readiness within the Saudi healthcare system.

### 4.2 Implementation evidence

Over the past decade, increasing evidence has demonstrated that successful implementation of rapid molecular point-of-care testing (mPOCT) depends not only on diagnostic performance but also on how testing is incorporated into healthcare delivery systems. Numerous implementation studies have shown that the greatest clinical benefit is achieved when molecular diagnostics are integrated into standardized clinical pathways that support immediate therapeutic decisions, patient triage, infection prevention, and antimicrobial stewardship rather than functioning as isolated laboratory investigations (24).

Emergency departments have emerged as one of the principal settings in which rapid molecular diagnostics provide operational value. Several studies have reported that availability of rapid molecular results during the initial patient encounter facilitates earlier clinical decision-making, more appropriate patient cohorting, and improved utilization of isolation facilities during seasonal respiratory outbreaks (12). Similarly, pediatric emergency departments and acute care services have demonstrated improvements in patient flow and more timely identification of viral respiratory infections when rapid molecular testing is incorporated into routine diagnostic pathways (26). These benefits are particularly relevant during periods of increased respiratory virus circulation, when healthcare systems experience substantial pressure on emergency services and inpatient capacity.

The impact of rapid molecular diagnostics on antimicrobial stewardship has also received considerable attention. Evidence suggests that molecular confirmation of viral pathogens may reduce diagnostic uncertainty and improve clinicians’ confidence in withholding or discontinuing unnecessary antibacterial therapy when supported by antimicrobial stewardship programs and evidence-based prescribing protocols (21). Nevertheless, systematic reviews consistently conclude that diagnostic testing alone rarely changes prescribing behavior unless accompanied by clinician education, institutional guidance, and stewardship interventions that translate laboratory results into appropriate clinical action (26). Accordingly, implementation strategies increasingly emphasize the integration of diagnostics with clinical governance rather than focusing exclusively on test availability.

Successful implementation also requires organizational readiness. Published implementation frameworks identify several recurring determinants of adoption, including workforce competency, availability of standardized operating procedures, clinical leadership, governance structures, reimbursement mechanisms, information technology integration, and continuous quality assurance (27, 28). Institutions with clearly defined testing algorithms and multidisciplinary implementation teams generally demonstrate more consistent utilization and greater clinician acceptance than organizations where testing is introduced without coordinated operational planning (27).

Economic evaluations have further suggested that rapid molecular diagnostics may offer health system benefits through reduced downstream healthcare utilization, optimized patient flow, improved infection control, and more efficient allocation of hospital resources (29). However, published findings remain heterogeneous because cost-effectiveness depends heavily on local disease epidemiology, healthcare organization, testing volume, reimbursement models, and baseline diagnostic practices. Consequently, implementation decisions should be informed by context-specific evaluations rather than assuming that economic findings from other healthcare systems are directly transferable to Saudi Arabia (30).

Collectively, current evidence indicates that rapid molecular POCT should be considered a complex health system intervention rather than a standalone diagnostic technology. Successful implementation requires coordinated investment in governance, workforce training, standardized clinical algorithms, digital infrastructure, and antimicrobial stewardship. These implementation principles provide an important framework for interpreting the stakeholder perspectives identified in the present study and for informing future national implementation strategies.

### 4.3 Saudi Arabia, Hajj and Umrah

Saudi Arabia presents a distinctive environment for the implementation of rapid molecular POCT because of its unique epidemiological profile, highly integrated healthcare system, and responsibility for hosting the world’s largest recurrent mass gatherings. Each year, millions of international pilgrims travel to the Kingdom to perform Hajj and Umrah, creating periods of intense population density that substantially increase opportunities for respiratory pathogen transmission and place considerable demands on healthcare services (31). Respiratory tract infections consistently represent one of the most frequently reported health conditions among pilgrims and remain a major focus of national surveillance, outbreak preparedness, and infection prevention programs (32, 33).

The epidemiology of respiratory infections during Hajj is characterized by the simultaneous circulation of multiple viral and bacterial pathogens, including influenza viruses, respiratory syncytial virus, SARS-CoV-2, rhinoviruses, seasonal coronaviruses, and other respiratory pathogens (34–37). Pilgrims often originate from geographically diverse regions with varying vaccination coverage, prior pathogen exposure, and underlying health status, increasing the complexity of clinical assessment and infection control during mass gatherings (38). Older adults and individuals with chronic diseases represent a substantial proportion of pilgrims, further increasing the risk of severe respiratory illness and healthcare utilization (33).

Rapid molecular diagnostics have become increasingly relevant within this setting because timely pathogen identification may facilitate early clinical triage, appropriate isolation measures, optimized patient flow, and more targeted therapeutic decision-making during periods of exceptionally high healthcare demand (39). Previous studies conducted during Hajj have demonstrated the feasibility of integrating molecular respiratory diagnostics into healthcare delivery and surveillance activities to support rapid detection of influenza, SARS-CoV-2, Middle East respiratory syndrome coronavirus (MERS-CoV), and other respiratory pathogens of public health importance (39). These diagnostic capabilities complement the Kingdom’s extensive public health surveillance infrastructure and contribute to national and international health security by strengthening early outbreak detection and situational awareness during mass gatherings.

Beyond Hajj, year-round Umrah pilgrimage results in continuous international population movement that further reinforces the importance of responsive respiratory diagnostic capacity throughout the healthcare system (40). Consequently, implementation of rapid molecular POCT within Saudi Arabia should be considered not only from the perspective of routine clinical care but also as an integral component of emergency preparedness, mass gathering medicine, infection prevention, and public health surveillance.

These considerations closely align with the objectives of Saudi Vision 2030, which emphasizes healthcare transformation, digital health integration, quality improvement, preparedness for emerging infectious diseases, and strengthening national public health capabilities (41). Within this strategic framework, rapid molecular POCT has the potential to support earlier diagnosis, standardized clinical pathways, antimicrobial stewardship, and more resilient healthcare delivery during both routine respiratory seasons and periods of exceptional healthcare demand associated with mass gatherings.

Taken together, the available evidence indicates that implementation of rapid molecular POCT in Saudi Arabia extends beyond adoption of a new diagnostic technology. Rather, it represents an opportunity to strengthen integrated respiratory surveillance, enhance clinical decision-making, improve healthcare system resilience, and support national preparedness for seasonal epidemics, emerging respiratory pathogens, and future public health emergencies.

## 5 Discussion

### 5.1 Principal findings

This mixed-methods implementation study integrated stakeholder perspectives, qualitative expert discussions, and published evidence to evaluate the perceived clinical value, implementation readiness, and health system considerations for rapid molecular point-of-care testing (mPOCT) for respiratory infections in Saudi Arabia. Rather than evaluating diagnostic performance or clinical outcomes, the study explored how healthcare professionals perceive the current implementation of rapid molecular diagnostics and identified priorities for strengthening their integration into routine clinical practice.

Several principal findings emerged. First, stakeholders consistently identified rapid turnaround time as the defining attribute of molecular POCT, emphasizing that its greatest perceived value lies in supporting clinical decisions during the initial patient encounter. Participants regarded emergency departments, triage areas, and other acute care settings as the environments in which rapid molecular diagnostics are most likely to influence patient management, reflecting the importance of timely microbiological information in respiratory care.

Second, implementation readiness was generally perceived to be moderate to high across participating institutions; however, substantial variability was observed in testing pathways, workflow integration, institutional guidance, and availability of standardized diagnostic algorithms. These findings suggest that implementation maturity differs across healthcare settings and that successful adoption depends not only on access to molecular platforms but also on organizational readiness, workforce education, and integration into established clinical workflows.

Third, educational needs, governance, reimbursement considerations, and variability in standard operating procedures emerged as the most frequently reported implementation challenges. Stakeholders consistently emphasized that sustainable implementation requires standardized diagnostic pathways supported by appropriate training, institutional leadership, and multidisciplinary collaboration. These observations reinforce the concept that rapid molecular POCT should be considered a healthcare system intervention rather than simply a diagnostic technology.

Finally, triangulation of quantitative survey findings, qualitative stakeholder discussions, and published evidence demonstrated substantial convergence across all three evidence sources. Speed of diagnosis, integration into clinical workflows, standardized testing algorithms, workforce competency, and diagnostic stewardship were consistently identified as the principal determinants of successful implementation. Collectively, these findings provide a context-specific framework that may inform future implementation strategies for rapid molecular POCT within Saudi Arabia and other healthcare systems with similar organizational structures.

### 5.2 Interpretation in the context of previous literature

The stakeholder perspectives identified in this study are broadly consistent with published implementation research demonstrating that the value of rapid molecular POCT extends beyond analytical test performance and depends largely on its ability to support timely clinical decision-making within routine healthcare workflows. International implementation studies have shown that rapid molecular diagnostics are most effective when results are available during the initial clinical encounter, enabling clinicians to incorporate microbiological evidence into decisions regarding treatment, patient disposition, infection prevention, and isolation measures (42, 43). The emphasis placed by stakeholders on rapid turnaround time and point-of-care deployment closely aligns with these observations and reinforces the importance of workflow integration as a prerequisite for realizing the potential benefits of molecular diagnostics.

Participants also consistently identified speed, diagnostic reliability, and workflow efficiency as the principal attributes contributing to the perceived value of rapid molecular POCT. Similar findings have been reported across emergency medicine, pediatric care, and acute respiratory services, where implementation studies have demonstrated that timely molecular diagnosis may facilitate more efficient patient management when incorporated into standardized clinical pathways (44, 45). However, it is important to recognize that the present study evaluated stakeholder perceptions rather than objective clinical outcomes. Consequently, the reported benefits should be interpreted as implementation priorities that are consistent with existing evidence rather than direct measures of clinical effectiveness.

Another important observation was the variability in implementation practices across participating institutions. Although many respondents reported the availability of respiratory testing algorithms and standard operating procedures, implementation remained heterogeneous, particularly regarding testing location, workflow integration, and institutional guidance. Similar implementation challenges have been described internationally, where governance structures, clinician engagement, workforce competency, and organizational readiness frequently exert greater influence on successful implementation than the diagnostic technology itself (46). These findings support the growing recognition that successful deployment of rapid molecular diagnostics requires coordinated implementation strategies that integrate technology with clinical governance and healthcare delivery systems.

Stakeholders also perceived rapid molecular POCT as having an important role within diagnostic stewardship and antimicrobial stewardship programs. Published studies have suggested that molecular confirmation of viral respiratory pathogens may reduce diagnostic uncertainty and support more appropriate antimicrobial prescribing when combined with evidence-based stewardship interventions (47, 48). Nevertheless, systematic reviews consistently emphasize that diagnostic testing alone is unlikely to change prescribing behavior in the absence of standardized clinical pathways, clinician education, and institutional stewardship programs (49). The perspectives expressed by participants in this study closely reflect this broader implementation literature by emphasizing education, governance, and standardized algorithms as essential components of successful implementation rather than relying solely on improved diagnostic capability.

Finally, the implementation barriers identified by stakeholders, including limited education, reimbursement challenges, variability in institutional guidance, and inconsistent integration into routine clinical workflows, are consistent with barriers reported across diverse healthcare systems. Rather than representing technology-specific limitations, these findings highlight common organizational challenges encountered during implementation of healthcare innovations. The convergence observed between stakeholder perspectives and published implementation evidence strengthens confidence that the priorities identified in this study are relevant not only to Saudi Arabia but also to other health systems seeking to expand the use of rapid molecular diagnostics for respiratory infections.

### 5.3 Implications for Saudi Arabia and mass gathering medicine

Saudi Arabia provides a distinctive setting for implementation of rapid molecular POCT because routine healthcare delivery must coexist with one of the world’s largest recurring mass gathering events. The annual Hajj pilgrimage and continuous year-round Umrah create periods of exceptionally high population density, international travel, and increased risk of respiratory pathogen transmission (50). These circumstances require diagnostic strategies that support both individual patient management and broader public health objectives, including surveillance, outbreak preparedness, infection prevention, and healthcare system resilience.

The stakeholder perspectives identified in this study suggest that rapid molecular POCT may be particularly valuable when incorporated into acute care pathways serving pilgrims and other high-risk populations (23). Participants consistently emphasized the importance of obtaining actionable diagnostic information during the initial clinical encounter, allowing clinicians to make timely decisions regarding patient triage, isolation, antiviral therapy, and hospital admission. During Hajj and Umrah, where large numbers of patients may present simultaneously with similar respiratory symptoms, timely molecular diagnosis has the potential to support more efficient patient cohorting and allocation of healthcare resources while strengthening public health surveillance.

The findings also reinforce the importance of standardized diagnostic pathways across the Saudi healthcare system. During mass gatherings, healthcare services are delivered through multiple temporary and permanent facilities involving multidisciplinary teams from different regions and institutions (51). Standardized national algorithms for respiratory molecular testing could reduce variability in clinical practice, improve consistency of diagnostic decision-making, and facilitate coordinated reporting of respiratory pathogens across healthcare facilities (31). Integration of rapid molecular diagnostics into electronic surveillance systems may further strengthen early outbreak detection and support national preparedness for emerging respiratory threats (23).

These implementation priorities align closely with the objectives of Saudi Vision 2030, which emphasizes healthcare transformation, digital health, quality improvement, and preparedness for public health emergencies (41). Rather than representing a standalone laboratory technology, rapid molecular POCT should be viewed as one component of a broader national strategy to strengthen respiratory disease surveillance, antimicrobial stewardship, emergency preparedness, and resilient healthcare delivery during both seasonal epidemics and mass gathering events.

### 5.4 Health system and policy implications

The findings of this study suggest several practical considerations for healthcare organizations and policymakers seeking to expand implementation of rapid molecular POCT. First, implementation strategies should prioritize integration of molecular diagnostics into standardized clinical pathways rather than focusing exclusively on acquisition of diagnostic platforms. Stakeholders consistently identified workflow integration, standardized patient selection criteria, and clearly defined clinical actions following test results as essential prerequisites for successful implementation.

Second, workforce development should represent a central component of implementation planning. Participants repeatedly highlighted the need for multidisciplinary education extending beyond technical operation of testing platforms to include appropriate test utilization, interpretation of molecular results, antimicrobial stewardship, and incorporation of diagnostic findings into routine clinical decision-making. Such educational initiatives should be supported by ongoing competency assessment and continuous quality improvement.

Third, organizational governance should be strengthened through development of harmonized national guidance and standardized operating procedures. Although many participants reported the availability of institutional testing algorithms, implementation remained heterogeneous across healthcare settings, suggesting opportunities for greater national coordination. Standardized guidance may improve consistency of practice while supporting diagnostic stewardship across primary care, emergency medicine, and hospital services.

Finally, stakeholders identified reimbursement and financial considerations as important barriers to wider implementation. Because the present study evaluated perceptions rather than economic outcomes, no conclusions regarding cost-effectiveness can be drawn. Nevertheless, the findings support the need for future Saudi-specific economic evaluations examining budget impact, cost-effectiveness, and resource utilization associated with implementation of rapid molecular POCT under routine clinical practice. Such evidence would complement implementation studies and inform national policy decisions regarding sustainable scale-up.

### 5.5 Strengths and limitations

This study has several important strengths. To our knowledge, it represents one of the first implementation-focused mixed-methods studies examining rapid molecular POCT for respiratory infections within the Saudi healthcare system. By integrating a structured stakeholder survey, multidisciplinary expert discussions, and evidence from the published literature, the study provides a comprehensive implementation perspective that extends beyond assessment of diagnostic performance alone. Triangulation of quantitative, qualitative, and published evidence strengthened the consistency of the findings and enabled identification of common implementation priorities across multiple sources of evidence. Furthermore, inclusion of participants representing clinical practice, laboratory medicine, point-of-care testing, and healthcare management allowed consideration of implementation from multiple professional perspectives.

The study also has several limitations. First, participants were recruited using a purposive sampling strategy through national scientific meetings held in Riyadh; therefore, the findings should not be considered statistically representative of all healthcare professionals or institutions within Saudi Arabia. Second, the relatively modest sample size limited analyses to descriptive summaries, and subgroup comparisons should be interpreted as exploratory observations rather than evidence of statistically significant differences. Third, the study evaluated stakeholder perceptions and reported implementation experiences rather than objective clinical, operational, microbiological, or economic outcomes. Consequently, no conclusions can be drawn regarding the effectiveness, cost-effectiveness, or clinical impact of rapid molecular POCT within Saudi healthcare settings. Fourth, qualitative findings were derived from expert discussions and thematic analysis rather than formal qualitative interviews designed to achieve thematic saturation. Finally, because implementation readiness and organizational practices may evolve over time, the findings represent stakeholder perspectives at the time of data collection and should be interpreted within the context of the rapidly changing respiratory diagnostic landscape.

Despite these limitations, the convergence observed across survey findings, qualitative themes, and published implementation evidence provides confidence that the priorities identified in this study represent important considerations for future implementation of rapid molecular POCT in Saudi Arabia. Future research should evaluate prospective implementation strategies using objective clinical, operational, and economic outcomes across multiple healthcare regions and during periods of routine respiratory activity as well as Hajj and Umrah seasons.

## 6 Conclusion

This implementation-focused mixed-methods study provides a comprehensive assessment of stakeholder perspectives, implementation readiness, and health system considerations for rapid molecular point-of-care testing (mPOCT) for respiratory infections in Saudi Arabia. By integrating a structured stakeholder survey, multidisciplinary expert discussions, and evidence from the published literature, the study offers a practical framework for understanding the opportunities and challenges associated with broader adoption of rapid molecular diagnostics within the Saudi healthcare system. Across all evidence sources, consistent priorities emerged. Stakeholders perceived rapid molecular POCT to be most valuable when integrated into time-sensitive clinical pathways that support immediate patient management, particularly in emergency departments, triage settings, and acute care services. Successful implementation was consistently linked not only to rapid diagnostic performance but also to standardized clinical algorithms, workforce education, organizational governance, and effective integration into routine healthcare workflows. At the same time, variability in implementation practices, reimbursement challenges, inconsistent institutional guidance, and educational needs were identified as important barriers requiring coordinated health system responses.

The findings have particular relevance for Saudi Arabia, where seasonal respiratory infections, continuous international travel associated with Umrah, and the annual Hajj pilgrimage create unique demands for timely respiratory diagnostics, surveillance, and outbreak preparedness. Within this context, rapid molecular POCT represents not only a diagnostic innovation but also a potential component of broader strategies aimed at strengthening antimicrobial stewardship, infection prevention, public health surveillance, and healthcare system resilience in alignment with Saudi Vision 2030. Because this study explored stakeholder perceptions rather than objective clinical, operational, or economic outcomes, its findings should be interpreted as implementation priorities rather than evidence of effectiveness or cost-effectiveness. Future multicenter implementation studies should evaluate the impact of rapid molecular POCT on patient outcomes, antimicrobial prescribing, healthcare resource utilization, and economic value across diverse healthcare settings, including routine respiratory seasons and mass gathering events. Overall, this study provides evidence-informed and stakeholder-informed implementation considerations that may support policymakers, healthcare leaders, and clinicians in developing standardized, sustainable, and context-specific strategies for responsible integration of rapid molecular POCT into respiratory care pathways in Saudi Arabia.
