# Supplementary material for: Speeding up respiratory diagnosis: clinical drivers, barriers, and system readiness for rapid molecular point-of-care testing in Saudi Arabia
Source: Front Public Health. 2026 Aug 11;14:1871669. doi: 10.3389/fpubh.2026.1871669 (PMC13503524; doi:10.3389/fpubh.2026.1871669)
Supplement: Supplementary file 1 [file Supplementary_file_1.pdf]

Stakeholder survey questionnaire

Title: Rapid Molecular Point-of-Care Testing (mPOCT) for Respiratory Infections – Stakeholder Survey

Purpose:
This survey aims to understand the current use, perceptions, and implementation status of rapid molecular point-of-care testing (mPOCT) for respiratory infections in healthcare settings.

Instructions:
Please answer the following questions based on your professional experience in your institution. Some questions allow multiple responses.

Section 1: Professional Background

1. What is your current professional role?

- Physician
- Laboratory specialist / microbiologist
- Point-of-care testing specialist
- Infection control specialist
- Healthcare administrator / policy stakeholder
- Other (please specify)

2. What type of institution do you work in?

- Ministry of Health (MOH) hospital
- Government institutional hospital
- Private sector hospital
- Academic / university hospital
- Other (please specify)

Section 2: Experience with Rapid Molecular Point-of-Care Testing (mPOCT)

3. How many years have you worked with rapid molecular point-of-care testing (mPOCT) platforms?

- I have not used mPOCT before
- <1 year
- 1–2 years
- 2–5 years
- 5 years

4. Does your hospital have a clear testing algorithm or protocol for respiratory pathogens (e.g., Influenza, RSV, COVID-19, Strep A)?

- Yes
- No
- I don't know

5. Where are respiratory samples typically tested in your hospital?

- Point-of-care device in the Emergency Department or triage area
- Point-of-care device in the hospital laboratory
- Sent to central laboratory within the hospital
- Sent to external reference laboratory
- Other (please specify)

6. How often does your department use rapid molecular point-of-care testing during the respiratory season?

- Never
- Rarely
- Weekly
- Several times per week
- Daily

Section 3: Clinical Use of Rapid mPOCT

7. What is the main reason you request or perform rapid molecular point-of-care testing for patients with respiratory symptoms?
(Select all that apply)

- Immediate treatment decision-making
- Support clinical diagnosis
- Patient management (e.g., bed designation or isolation)
- Emergency department workflow optimization
- Infection control measures
- Reliable confirmation of respiratory pathogens
- Other (please specify)

8. For which conditions do you prefer using rapid molecular point-of-care testing over traditional laboratory testing?
(Select all that apply)

- Influenza A/B
- COVID-19
- Respiratory Syncytial Virus (RSV)
- Group A Streptococcus
- Other respiratory pathogens
- None

__Section 4: Implementation status__

9. To what degree is rapid molecular point-of-care testing implemented to its full potential in your hospital?

Scale from **0 to 10**

- 0 = Not implemented at all
- 10 = Fully implemented

Score: ______

10. If the score is less than 10, what are the main barriers preventing full implementation? *(Select all that apply)*

- Lack of education or training
- Lack of supportive policies or governance
- Financial or reimbursement constraints
- Not included in national or hospital guidelines
- Limited availability of testing platforms
- Perception that better alternatives exist
- Operational workflow challenges
- Other (please specify)

11. In which clinical areas do you see the greatest potential for further implementation of rapid mPOCT?

- Emergency department
- Acute care wards
- Outpatient clinics
- Intensive care units
- Primary care
- Other (please specify)

Section 5: Standard Operating Procedures and Guidelines

12. Are there standard operating procedures (SOPs) in your hospital for rapid molecular point-of-care testing?

- Yes
- No
- I don’t know

13. If yes, what aspects are covered in these SOPs?
(Select all that apply)

- Who should perform the test
- Which patients should be tested
- How the test should be performed
- When the test should be used in the patient pathway
- Why rapid mPOCT should be used
- Other (please specify)

14. Are there national or hospital guidelines supporting the use of rapid molecular point-of-care testing for respiratory infections?

- Yes – national guidelines
- Yes – hospital guidelines
- No
- I don’t know

Section 6: Perceptions and Value

15. What is the general opinion about rapid molecular point-of-care testing in your country or region for diagnosing respiratory infections?

Rate from **1 to 10**

- 1 = Very negative
- 10 = Very positive

Score: ______

16. What are the main reasons hospitals in your country use rapid molecular point-of-care testing?
(Select all that apply)

- Speed of testing
- Reliable results
- Accurate results
- Patient management and workflow efficiency
- Antimicrobial stewardship benefits
- Support during high-demand respiratory seasons
- Cost-effectiveness
- Other (please specify)

17. What challenges may limit the adoption of rapid molecular point-of-care testing in your country?
(Select all that apply)

- Lack of supportive policies
- Lack of education or training
- Financial constraints
- Limited infrastructure
- Negative perception of POCT
- Availability of alternative diagnostic methods
- Other (please specify)

Section 7: Operational considerations

18. What is the ideal turnaround time for rapid molecular point-of-care testing (from sample collection to result)?

- <15 minutes
- <30 minutes
- <60 minutes
- 60 minutes

19. What is the most important benefit of rapid molecular point-of-care testing in your hospital?
(Select up to three)

- Speed of testing
- Reliable / accurate results
- Patient management and workflow efficiency
- Antimicrobial stewardship
- Cost-effectiveness
- Support during high-demand respiratory seasons
- Other (please specify)

20. How do you perceive the cost-to-clinical utility ratio of rapid molecular point-of-care testing compared with other diagnostic methods?

- Better than standard lab-based PCR
- Better than lateral flow testing
- No significant difference
- Missing

Section 8: Clinical volume

21. Approximately how many patients with suspected respiratory infections present to your department during a respiratory season?

- <500
- 500–1000
- 1000–5000
- 5000
- Not sure

22. Approximately how many respiratory diagnostic tests are performed in your department per week during respiratory season?

- <50
- 50–100
- 100–500
- 500
- Not sure
