# Supplementary material for: Speeding up respiratory diagnosis: clinical drivers, barriers, and system readiness for rapid molecular point-of-care testing in Saudi Arabia
Source: Front Public Health. 2026 Aug 11;14:1871669. doi: 10.3389/fpubh.2026.1871669 (PMC13503524; doi:10.3389/fpubh.2026.1871669)
Supplement: Supplementary file 2 [file Supplementary_file_2.pdf]

Supplementary Table S1. Availability of respiratory diagnostic algorithms for Flu, RSV, COVID-19, and Strep A (N = 35)

Availability of a clear testing algorithm	n	%
Yes	26	74.3
No	7	20.0
I do not know	2	5.7

Footnote: Algorithm availability reflects participant perception of whether an institutional algorithm exists for appropriate selection of Flu, RSV, SARS-CoV-2, and Strep A testing. Abbreviations: RSV, respiratory syncytial virus.

Supplementary Table S2. Testing workflow location for rapid molecular POCT (mPOCT) (N = 35)

Testing workflow location	n	%
ED/triage/wards (true POC setting)	16	45.7
POC platform located in laboratory	9	25.7
Sample sent to central laboratory	6	17.1
Sample sent to external/referral laboratory	4	11.4

Footnote: "True POC setting" aligns with workflows where results can directly influence immediate clinical decisions within the care area (e.g., emergency department or wards). Abbreviations: ED, emergency department; POC, point-of-care.

Supplementary Table S3. Preferred respiratory pathogens and clinical conditions for rapid mPOCT (Multiple Responses Allowed) (N = 35)

**Preferred condition/pathogen**	**n**	**%**
All are relevant	18	51.4
Respiratory syncytial virus (RSV)	13	37.1
Influenza A and B	12	34.3
SARS-CoV-2 (COVID-19)	11	31.4
Group A Streptococcus (Strep A)	7	20.0

Footnote: Participants were able to select more than one condition, therefore percentages sum to more than 100%.
Abbreviations: POCT, point-of-care testing; RSV, respiratory syncytial virus.

Supplementary Table S4. Perceived Benefits and Value Drivers of Rapid mPOCT (Multiple Responses Allowed) (N = 35)

**Reported benefit/value driver**	**n**	**%**
Speed of testing	30	85.7
Patient management and workflow efficiency	22	62.9
Reliable/accurate results	19	54.3
Antimicrobial stewardship	16	45.7
Support during high-demand periods	14	40.0
Cost-effectiveness	11	31.4
Patient satisfaction	1	2.9
Tool to convince patients	1	2.9

Footnote: Participants could select multiple responses. The two lowest-frequency items were retained as they reflect specific real-world perceptions and communication needs.
Abbreviations: POCT, point-of-care testing.

Supplementary Table S5. Perceived cost–utility ratio of rapid mPOCT compared with other diagnostic methods (N = 35)

**Cost–utility perception**	**n**	**%**
Better than standard lab-based PCR	15	42.9
Better than lateral flow testing	12	34.3
No significant difference	7	20.0
Missing	1	2.9

Footnote: "Standard lab-based PCR" refers to conventional centralized laboratory PCR with longer turnaround time.

Abbreviations: PCR, polymerase chain reaction; POCT, point-of-care testing.
